# Dietary Genistein Alleviates Lipid Metabolism Disorder and Inflammatory Response in Laying Hens With Fatty Liver Syndrome

**DOI:** 10.3389/fphys.2018.01493

**Published:** 2018-10-24

**Authors:** Zengpeng Lv, Kun Xing, Guang Li, Dan Liu, Yuming Guo

**Affiliations:** State Key Laboratory of Animal Nutrition, College of Animal Science and Technology, China Agricultural University, Beijing, China

**Keywords:** genistein, hens, fatty liver, fatty acids, inflammation

## Abstract

This study investigated the molecular mechanism underlying the effect of dietary genistein (GEN) on fatty liver syndrome (FLS) in laying hens. Hens in the control group (CG) were fed a high-energy and low-choline (HELC) diet to establish the FLS model. The livers of the FLS hens were friable and swollen from hemorrhage. Hepatic steatosis and inflammatory cell infiltration were present around the liver blood vessels. Hens in the low-genistein (LGE) and high-genistein (he) groups were fed GEN at 40 and 400 mg/kg doses, respectively, as supplements to the HELC diet. GEN at 40 mg/kg significantly increased gonadotropin-releasing hormone (GnRH) mRNA expression in the hypothalamus, the serum estrogen (E2) level, and the laying rate, whereas 400 mg/kg of GEN decreased GnRH expression and the laying rate without significantly affecting E2, suggesting that high-dose GEN adversely affected the reproductive performance. Either high- or low-dose GEN treatment could alleviate metabolic disorders and inflammatory responses in FLS hens. GEN significantly decreased the serum ALT, creatinine, triglyceride (TG), total cholesterol (TC), and free fatty acid (FFA) levels. Accordingly, the TG and long-chain fatty acid (LCFA) levels, including long-chain saturated fatty acids (LSFAs) and monounsaturated fatty acids (MUFAs), and the n-6:n-3 polyunsaturated fatty acid (PUFA) ratio in the liver were reduced after the GEN treatments, whereas the levels of C22:0, n-3 family fatty acids, C20:3n6, and C20:4n6 were increased. These results indicated that dietary GEN downregulated the expression of genes related to fatty acid synthesis [sterol regulatory element-binding protein 1 (SREBP1c), liver X receptor alpha (LXRα), fatty acid synthase (FAS), and acetyl coenzyme A synthetase (ACC)] and the fatty acid transporter (FAT). Furthermore, GEN treatments upregulated the transcription of genes related to fatty acid β-oxidation [peroxisome proliferator-activated receptor (PPAR)α, PPARδ, ACOT8, ACAD8, and ACADs] in the liver and reduced PPARγ and AFABP expression in abdominal fat. Dietary GEN alleviated inflammatory cell infiltration in the livers of FLS hens and downregulated TNF-α, IL-6, and IL-1β expression. Moreover, GEN treatment increased SOD activity and decreased malondialdehyde activity in the liver. In conclusion, GEN supplementation in the feed inhibited fatty acid synthesis and enhanced β-oxidation in the liver through the PPAR–ACAD/ACOT and PPAR–LXRα–SREBP1c–ACC/FAS/FAT pathways. Dietary GEN alleviated metabolic disorder and inflammation in the FLS hens by improving the antioxidant capacity and fatty acid profile.

## Introduction

Fatty liver syndrome is one of the most common metabolic diseases in laying hens, especially during the late laying period. The FLS can significantly decrease egg production and induce sudden death, resulting in major economic losses for the poultry industry. Marked fatty degeneration and fat deposition occur in the parenchymal cells of the fatty liver (Giulio et al., 2003). The widely accepted “two-hit” theory indicates that disordered fatty acid metabolism is a common element of the FLS (Erickson, 2009). Thus, we speculated that high amounts of FFAs in the livers of hens during the late egg-laying period could destroy the structure of biofilms through free radicals. Excessive saturated fatty acids (SFAs) have been reported to not only have direct toxic effects on hepatocytes but to also indirectly cause cell damage (Kohjima et al., 2005). Furthermore, increased plasma FFA levels can activate toll-like receptor 4 on the surface of macrophages and adipocytes, thereby inducing an inflammatory response in the fatty liver (Yuan, 2001). Activated Kupffer cells in the liver can secrete large amounts of cytokines, including TNF-α, IL-1, IL-6, and IL-8 (Rutkowski et al., 2009). TNF-α promotes FFA secretion in peripheral tissue and fatty acid synthesis in the liver; IL-1 and IL-6 can likewise hinder the transport and secretion of TGs (Navasa et al., 1998).

The FLS can damage hepatocyte mitochondria and thus influence β-oxidation of fatty acids (Wei et al., 2008). Moreover, peroxisome proliferator-activated receptors (PPARs), which regulate β-oxidation and energy metabolism, have a close relationship with FLS (Rao and Reddy, 2004; Zhou et al., 2008). Previous studies have shown that activated PPARs can form heterodimers with the nuclear retinoid X receptor and regulate the transcription of targeted proteins (fatty acid-binding proteins, LPL, phosphoenolpyruvate carboxykinase, stearoyl-coenzyme A desaturase, and the β-oxidation enzyme system) by binding to the PPAR response element (Hwang et al., 1997; Uppenberg et al., 1999). Overexpression of PPARδ in brown adipose tissue of mice upregulates the transcriptional levels of genes related to TG hydrolysis, fatty acid oxidation, and uncoupled oxidative phosphorylation, including acyl-coenzyme A oxidase, muscle carnitine palmityl transferase-1, long-chain and very long-chain acyl-coenzyme A dehydrogenase, and uncoupling proteins (Wang et al., 2003).

Genistein is a type of isoflavone (ISO) widely found in leguminous plants, especially in soya bean and its products. This ISO is structurally similar to E2, allowing it to bind to the E2 receptor. GEN is considered a safe environmental E2. The average human dietary intake of GEN is 2 mg/kg body weight/day (Setchell et al., 1998). Studies have revealed beneficial effects of ISO with regard to hypercholesterolemia, obesity, diabetes mellitus, and menopausal symptoms (Mazur et al., 1998; Merzdemlow et al., 2000; Hirota et al., 2010; Khan et al., 2011; Kurita et al., 2016). The polyhydroxyphenol nature of GEN enables it to eliminate a variety of reactive oxygen species (ROS), thereby reducing oxidative damage in the skeletal muscle of broilers (Jiang et al., 2007). However, ISO metabolism and sensitivity varies among animals. Significant differences are noted in the amount of GEN in the livers of old hens and male broiler chickens after GEN treatment (Stevenson et al., 2014). Dietary GEN at a dose of 800 mg/kg feed has been reported to significantly increase the laying performance of quails (Akdemir and Sahin, 2009). Moreover, adding GEN to the diet of hamsters can markedly relieve oxidative stress caused by lipid peroxidation and lower the serum LPL level (Fang et al., 2004). GEN can activate PPARα and PPARγ simultaneously and promote β-oxidation (Mezei et al., 2003). Several studies have indicated that ISO can exert anti-inflammatory activity. Dietary GEN at a dose of 20–80 mg/kg feed improves the growth performance and immunity of broiler chickens (Chun et al., 2008; Gudbrandsen et al., 2009). LCFAs can induce lipid peroxidation and hepatocyte damage through interactions among TNF-α, PPARα, CYP2E1, leptin, and UCP2 (Kohjima et al., 2005). However, PUFAs exert a negative regulatory effect on pathogenesis of the fatty liver. PUFAs can inhibit the expression of pro-inflammatory factors and increase endogenous anti-inflammatory factors (Stulnig, 2003). n-3 PUFAs were reported to decrease fat accumulation in the livers of both obese rats and patients with nonalcoholic fatty liver disease (Capanni et al., 2006). It is pivotal to investigate the effects of GEN on the fatty acid profile in the liver of hens with FLS. Therefore, with this study, we aim to clarify the molecular mechanism whereby dietary GEN relieves FLS in laying hens through interactions among lipid metabolism, inflammatory response, and antioxidation.

## Materials and Methods

### Birds and Husbandry

The animal experimental procedures were approved by the China Agriculture University Animal Care and Use Committee, Beijing, China. A total of 405 80-week-old JINGFEN 1 laying hens of similar weight (1870.9 ± 202.1 g) were randomly allotted to three treatments, with nine replicates of 15 birds each. The hens were raised in ladder cages with three birds each. During the two-week acclimation period, hens were exchanged between the pens to form replicates of similar initial mean BW and egg production. The formal experimental period was from 82 to 90 weeks of age. According to the Nutrient Requirements of Poultry (NRC, 1994) and Zhang et al. (2018), the control group (CON) was fed a HELC basal diet to establish a pathological fatty liver model (Table 1). The LGE and HGE groups were fed a diet identical to that of the CON group, but the feed was supplemented with GEN at 40 and 400 mg/kg diet, respectively. The GEN was a synthetic product with 99.9% purity from Kai Meng Co. (Xi An, China) Chemical Plant. All groups were fed the experimental diets for 64 days. The experimental diets and water were provided *ad libitum*. Eggs were collected daily, and the weight and number of all the eggs produced were determined for each replicate. Food intake was recorded at 1-week intervals. The room temperature was set at 25 ± 3°C, and the light was controlled on a light:dark cycle of 16:8 h. All procedures, as well as the care, housing, and handling of the animals, were conducted according to accepted commercial management practices. The animal experiment was conducted in the China Agricultural University Poultry Facility (Zhuozhou, China).

**Table 1:** Composition and nutrient levels of diet.

Ingredient	% of diet, weeks 80–90
Corn	65
Soybean meal	3.5
Corn protein	3.917
Gossypol-free cottonseed protein	10
Limestone	8.5
Soybean oil	2.011
Dicalcium phosphate	1.715
NaCl	0.35
Zeolite	2
Trace mineral premix^a^	0.3
Choline chloride (50%)	0.12
Mycotoxin adsorbent	0.1
DL-methionine	0.09
Vitamin premix^b^	0.035
Santoquin	0.03
Phytase	0.015
4% flavomycin	0.015
Lysine⋅HCl (8%)	0.258
Threonine	0.024
Tryptophan	0.085
Wheat middlings	1.935
Total	100
Available metabolic energy (MC/kg)	2.91
Crude protein (%)	16.1
Calcium (%)	3.5
Total phosphorus (%)	0.6
Available phosphorus (%)	0.48
Methionine (%)	0.35
Lysine (%)	0.75
Met + Cys (%)	0.6
Threonine (%)	0.54
Tryptophan (%)	0.16

*^a^The trace mineral premix supplied the following per kilogram of complete feed: copper, 8 mg; zinc, 75 mg; iron, 80 mg; manganese, 100 mg; selenium, 0.15 mg; iodine, 0.35 mg. ^*b*^The vitamin premix supplied the following per kilogram of complete feed: vitamin A, 12,500 IU; vitamin D3, 2,500 IU; vitamin K3, 2.65 mg; vitamin B1, 2 mg; vitamin B2, 6 mg; vitamin B12, 0.025 mg; vitamin E, 30 IU; biotin, 0.0325 mg; folic acid, 1.25 mg; pantothenic acid, 12 mg; niacin, 50 mg*.

At 90 weeks of age, two chicks per replicate, with body weights close to the average, were selected after 10 h of feed deprivation. One blood sample was collected from the wing vein of each chick in vacuum blood collection tubes. The serum was separated out by centrifugation at 3000 ×*g* for 15 min and stored at -20 °C until it was used for the measurement of hormones and biochemical indices. Additional blood samples were collected from the wing vein into vacuum blood collection tubes (with EDTA) for routine blood tests. Then, two chickens from each replicate were killed by decapitation. The liver, the spleen, and the abdominal fat were measured to calculate the organ indices. Tissue samples from those three locations were collected, frozen in liquid nitrogen, and kept in a freezer (-80°C) for measurements of gene expression, antioxidative indices, and LCFAs.

### Radioimmunoassay for Serum Hormone Concentrations

The serum levels of E2 were measured using commercial double-antibody radioimmunoassay kits purchased from Shanghai Institute of Biological Products. The interassay coefficient of variation was 10%.

### Determination of Antioxidant Enzyme Activity and Malondialdehyde (MDA) Levels

The formation of MDA was used as an indicator of lipid peroxidation *via* the thiobarbituric acid assay (MDA detection kit A003, Jiancheng Bioengineering Institute, Nanjing). Glutathione peroxidase (GSH-Px), catalase (CAT), and superoxide dismutase (SOD) activity were determined using kits from Nanjing Jiancheng Bioengineering Institute (CAT detection kit A0071-1, SOD detection kit A001-3, GSH-Px detection kit A005). The protein concentrations of the samples were measured using the Bradford method (Bradford, 1976).

### Serum Biochemical Indices and Routine Blood Tests

Serum biochemical indices, including GPT, glutamic-oxaloacetic transaminase (GOT) and creatinine (CRE), as well as TGs, FFAs, TC, and very low-density lipoprotein (VLDL) were measured using assay kits (Unicel DXC 800, CA, United States). VLDL was examined using commercially available colorimetric diagnostic kits (H249, Nanjing Jiancheng Bioengineering Institute, China). Routine blood tests were conducted for red blood cells (RBC), hematocrit (HCT) and hemoglobin (HGB), platelets (PLT), procalcitonin (PCT), LUC (large unstained cells), basophils (BASO), and white blood cells (WBC) using assay kits (Sysmex KX-21 N automatic blood analyzer, Kobe, Japan).

### Pathological Observation

Tissue blocks were fixed in 10% formalin. After 72 h, liver samples of suitable size were taken for routine paraffin embedding and hematoxylin and eosin (HE) staining. Light microscopy (LEICA DMI6000 B) was used to observe and record histopathological changes.

### Serum Antibody and Immunoglobulin Levels

The serum antibody titers against Newcastle disease (ND) and four avian influenza viruses (RE-6, RE-7, RE-8, and H9) were determined using a commercial ELISA kit (IDEXX Laboratories Inc., Westbrook, ME, United States) according to the manufacturer’s protocol.

### Long-Chain Fatty Acid (LCFA) Analysis

We first used a vacuum freeze-drying machine (CA301/801, SANYO, Japan) to dry the liver samples. Then, lipids were extracted for the subsequent LCFA analysis according to the method of Bligh and Dyer (Bligh and Dyer, 1959). The methyl esters of the LCFAs from the lipid extract were transesterified with hydrochloric acid (HCl) in methanol according to the method described by Ichihara and Fukubayashi (2010). LCFAs were quantified using an Agilent Technologies 7890A Gas Chromatograph (Santa Clara, CA, United States) with a flame ionization detector. The LCFAs were separated on a 112-88A7 HP-88 capillary column (100 m × 0.25 mm, 0.2 μm) using helium (He) as a carrier gas at a flow rate of 105 mL/min. The samples were injected at a starting oven temperature of 175°C; the injector temperature was 250°C, and the detector temperature was 280°C. The oven temperature was programmed to increase from 175 to 220°C. Fatty acids were identified by their retention time compared with reference fatty acid standards (Supelco, FAME Mix, Sigma-Aldrich) and are expressed in milligrams per gram of total fatty acids in the liver.

### Quantitative Reverse Transcription Polymerase Chain Reaction (qRT-PCR) Analysis

Total RNA was isolated from liver samples using TRIzol reagent (Invitrogen, Carlsbad, CA, United States) according to the manufacturer’s protocol. cDNA synthesis was performed using a PrimeScript RT reagent kit with gDNA Eraser (TaKaRa, Dalian, Liaoning, China) according to the manufacturer’s instructions. One-step real-time RT-PCR was performed using SYBR Premix Ex Taq^TM^ (TaKaRa, Dalian, Liaoning, China) in a real-time PCR machine (ABI7500; Applied Biosystems, Carlsbad, CA, United States) following the manufacturer’s guidelines. The primer pairs used are shown in Table 2. The housekeeping gene β-actin was utilized as an internal control. Relative mRNA expression levels of each target gene were normalized to the control using the 2^-ΔΔCT^ method. As previously described, total RNA was treated with deoxyribonuclease, reverse transcribed, and amplified by qPCR with oligonucleotide primers specific to chickens. The net intensity ratios of target genes to GAPDH were used to represent the relative levels of target gene expression. The means of two replicates were used for statistical analysis.

**Table 2:** Primers used for quantitative real-time PCR analysis.

Gen name	5^′^ primer	Prod side
SREBP1c	F: GCCCTCTGTGCCTTTGTCTTC	122
	R: ACTCAGCCATGATGCTTCTTC	
FAS	F: TGGACGAGTGTATGAGATGTCG	138
	R: CGCAATGTTCACACCGAGA	
LXRα	F: CAAAGGGAATGAATGAGC	145
	R: AGCCGAAGGGCAAACAC	
PPARα	F: TCCTTCCCGCTGACCAAA	112
	R: TCCTGCACTGCCTCCACA	
PPARγ	F: CGAGGAGTCTTCCAACTC	135
	R: CCTGATGGCATTATGTGA	
PPARδ	F:TGAATGACCAAGTGACTCTGCTGAAG	320
	R: CAGTGCTCGGAGGATGTTGTCTTG	
ACOT8	F: CCACTCGCTTCACTGCTACTTCG	322
	R: TGGCACATCTTCAGCTTGGATCTTG	
FAT	F: ACTGGGAAGGTTACTGCGATT	119
	R: TCACGGTCTTACTGGTCTGGT	
ACC	F: AATGGCAGCTTTGGAGGTGT	114
	R: TCTGTTTGGGTGGGAGGTG	
HMGCoAS	F: AGCAAATGGTGTTTTCAGTGG	117
	R: AGAAGCAAGGCAACCGTAGAC	
ACAD8	F: GATTGTCACGTCTCGATACCTCCATC	138
	R: CCTGTGCCTCTGTTCCTCATTGC	
ACADs	F: GCTCTGGACTGTGCTGTGGATTATG	284
	R: CGTTCTGCTGGCATCTCTGTCAC	
Wnt5a	F: ACTTGGCAGCACAATGGCTT	96
	R: GACCACCAAGAGCTGGCTTC	
IL-1β	F: ACTGGGCATCAAGGGCTA	104
	B: GGTAGAAGATGAAGCGGGTC	
IL-8	F: ATGAACGGCAAGCTTGGAGCTG	122
	R: TCCAAGCACCTCTCTTCCATCC	
NF-κB	F: GTGTGAAGAAACGGGAACTG	118
	R: GGCACGGTTGTCATAGATGG	
AFABP	F: CAGCATCAATGGTGATGT	134
	R: ACAGTCTCTTTGCCATCC	
TNF-α	F: GAGCGTTGACTTGGCTGTC	121
	R: AAGCAACAACCAGCTATGCAC	
IFN-γ	F: AGCTGACGGTGGACCTCA	116
	R: GGCTTTGCGCTGGATTC	
TCF3	F: TGCTCCACAACCATGTCACACTTC	146
	R: TGTTCTCCTCATCCTCCTTCTCTTCC	
GAPDH	F:TGCTGCCCAGAACATCATCC	120
	R:ACGGCAGGTCAGGTCAACAA	

*F represent forward, R represent reward*.

### Statistical Analysis

The results were expressed as the mean ± SD or mean ± SEM (for gene expression), and differences were considered significant when *P* < 0.05 as tested by ANOVA in SPSS 11.0 for Windows.

## Results

### Assessment of Production Performance

**FIGURE 1:**
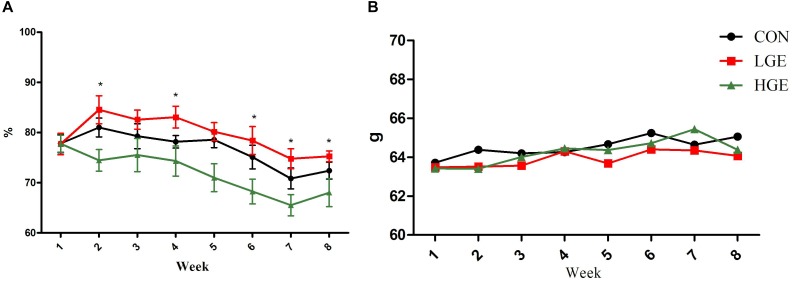
**(A,B)** Represent the effects of GEN supplementation on the egg-laying rate and average egg weight of laying hens during the late egg-laying period, respectively. Mean values with (^∗^) differ significantly between the control and GEN-treated hens (*n* = 9 replicates).

In the present study, dietary GEN at the 40 mg/kg dose increased the egg-laying rates of laying hens at weeks 1, 4, 6, 7, and 8. However, the 400 mg/kg supplementation level decreased the egg-laying rates during the whole experimental period (*P* < 0.05, Figure 1A). As shown in Figure 1B, the egg weight of hens in the LGE group was lower than that of the CON group, but the difference was not significant. In addition, High-dose GEN treatment increased feed/egg ratio compared with other two groups (*P* = 0.063, Table 3). Dietary GEN had no significant effects on the feed intake of laying hens in this experiment (Table 3).

**Table 3:** Effect of dietary GEN on feed/egg ratio, daily feed intake, organ indices, and liver scores in hens.

Treatment	CON	LGE	HGE	*P*-value
Daily feed intake (g)	103.4 ± 4.7	100.6 ± 4.3	104.0 ± 3.2	0.187
Feed/egg ratio	2.14 ± 0.11	2.09 ± 0.03	2.25 ± 0.06	0.063
Abdominal fat rate (%)	6.85 ± 2.25a	4.43 ± 1.25b	4.58 ± 1.78b	0.001
Liver index (%)	2.40 ± 0.28a	2.18 ± 0.24b	2.15 ± 0.25b	0.026
Spleen index (%)	0.104 ± 0.025	0.092 ± 0.023	0.084 ± 0.016	0.016
Clinical score of liver	4.00 ± 0.24a	2.10 ± 0.42b	2.51 ± 0.53b	<0.001
Pathological score of liver	2.4 ± 0.5a	0.3 ± 0.3b	0.4 ± 0.4b	<0.001

*Data represent the mean ± SD of values (*n* = 9 replicates, 2 samples each replicate). Mean values without a common identifier (a and b) differ significantly (*P* < 0.05)*.

### Levels of Hormones Involved in the Hypothalamic–Pituitary–Gonadal (HPG) Axis

The effects of GEN on the level of E2 in the serum of chickens are presented in Table 4. The level of E2 was significantly higher in the LGE group than in the CON group (*P* < 0.05), while the 400 mg/kg supplementation level had no significant effects on E2. Reproductive performance is mediated by the HPG axis; thus, we measured the mRNA expression of HPG axis-related genes using qPCR. The transcriptional level of GnRH in the hypothalamus was significantly upregulated in the LGE group (*P* < 0.05) compared with that in the CON group; the 400 mg/kg supplementation significantly downregulated GnRH mRNA expression (Figure 3, *P* < 0.05), which might be directly responsible for the different effects of the two doses on the laying performance.

**Table 4:** Effect of dietary GEN on the serum metabolic indices and routine blood indices of hens.^a^

Treatment	CON	LGE	HGE	*P*-value
Serum metabolic indices^1^				
GPT (U/L)	4.79 ± 1.12	3.83 ± 0.76	4.16 ± 0.74	0.069
GOT (U/L)	179.2 ± 42.4	151.3 ± 38.1	163.8 ± 25.8	0.198
TC (mmol/L)	4.40 ± 1.70a	2.63 ± 0.26b	2.65 ± 0.63b	0.012
TG (mmol/L)	17.0 ± 3.8a	11.1 ± 1.0b	11.7 ± 2.3b	0.001
CRE (μmol/L)	110.6 ± 22.2a	86.4 ± 5.9b	90.0 ± 9.4b	0.015
FFAs (μmol/L)	410.3 ± 28.5	388.0 ± 11.5	385.3 ± 16.0	0.070
E2 (pg/mL)	59.46 ± 8.30b	73.26 ± 6.91a	64.01 ± 7.23b	0.005
VLDL (mmol/L)	1.80 ± 0.22b	2.14 ± 0.30a	2.07 ± 0.29a	0.031
Routine blood indices^2^				
RBC (×10^12^ cells/L)	2.54 ± 0.23b	2.62 ± 0.21ab	2.70 ± 0.14a	0.041
HGB (g/L)	102.4 ± 10.6	107.2 ± 5.9	107.1 ± 8.0	0.121
PLT (×10^9^ cells/L)	98.4 ± 22.6	111.4 ± 34.2	124.3 ± 43.2	0.053
LUC (%)	1.84 ± 1.73	1.23 ± 0.85	1.05 ± 0.52	0.051
BASO (%)	0.74 ± 0.33b	1.06 ± 0.52a	0.75 ± 0.38b	0.025
WBC (×10^9^ cells/L)	44.8 ± 11.8	48.4 ± 12.2	53.0 ± 9.1	0.070

*^a^Serum samples were obtained from the wing vein (*n* = 9 replicates, 2 samples each replicate). ^*2*^Routine blood indices were measured in anticoagulant-treated blood from the wing vein. Data represent the mean ± SD of values. Mean values without a common identifier (a and b) differ significantly among the three groups (*P* < 0.05). ^*1*^Serum metabolic indices include GPT, glutamic-pyruvic transaminase; GOT, glutamic-oxalacetic transaminase; TC, total cholesterol; TG, triglyceride; CRE, creatinine; FFAs, free fatty acids; E2, estrogen; VLDL, very low-density lipoprotein; RBC, red blood cells; HGB, hemoglobin; PLT, platelets; LUC, large unstained cells; BASO, basophils; WBC, white blood cells*.

### Hepatic Scores, Statistics, and Organ Indices

**FIGURE 2:**
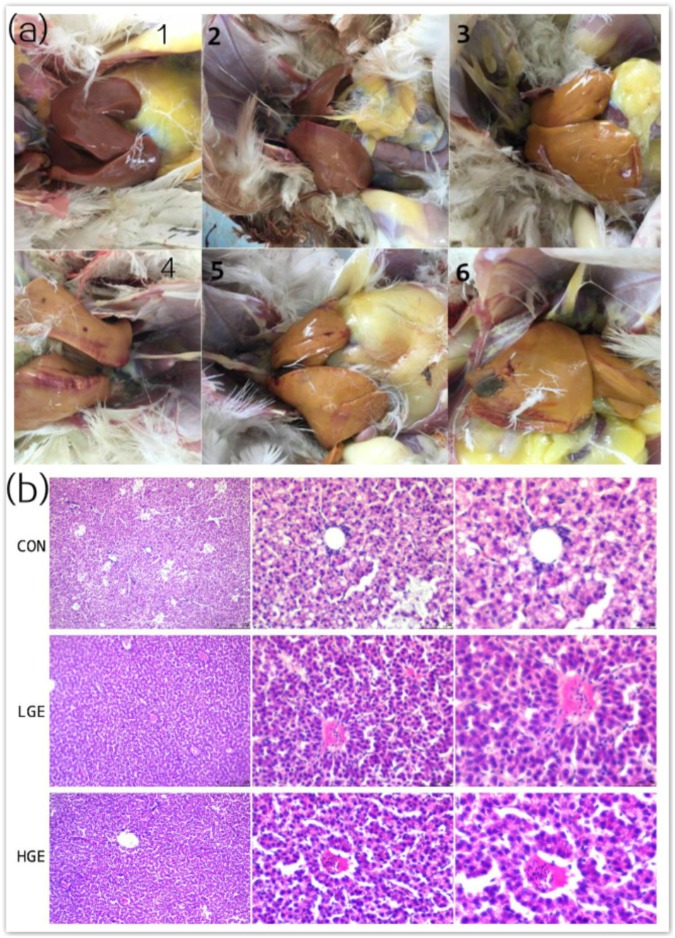
**(a)** Clinical scores for chicken livers were divided into six levels representing different degrees of FLS. **(b)** Chicken livers were stained with HE, viewed under a microscope (100×, 400×, and 600×), and assigned pathological scores representing the degree of FLS. The categorization scheme is shown in Table 5.

As is shown in Figure 2a, we classified the fatty liver severity into six levels according to the hepatic clinical scores. We defined levels 3 to 6 as the FLS. As shown in Table 4, the mean hepatic clinical score of the hens fed the control HELC diet was 4, and the livers of the CON group were enlarged and appeared yellowish or yellowish brown in color. The livers presented severe symptoms including rupture, bleeding, and friable texture. When the tissue was cut with a knife, fat drops clung to the surface of the knife. There were large amounts of fat deposited in the abdomen, subcutaneous tissue, pericardium, stomach, and mesentery. The clinical categorization scheme used for assessing fatty liver is shown in Table 5. The hepatic clinical scores of the LGE and HGE group were significantly lower than that of the CON group (*P* < 0.05), indicating the favorable, anti-FLS effect of GEN. In terms of histological changes, we observed hepatocyte tumefaction and focal necrosis in the CON group (Figure 2b). The hepatocytes of the CON group contained fat vacuoles of various sizes, which pushed the nuclei aside. In addition, the structures of the hepatic sinusoids were not clear. We also observed some inflammatory cell infiltration around the blood vessels. The hepatic pathological scores of the LGE and HGE groups were significantly decreased compared with those of the CON group. The sizes and shapes of the cells were normal; we did not detect any signs of hepatocyte tumefaction, granule denaturation or vacuolation. Furthermore, GEN treatment decreased the abdominal fat rate and the liver and spleen organ indices of laying hens during the late egg-laying period (Table 3, *P* < 0.05).

**Table 5:** The categorization scheme of pathological scores of fatty liver.^a^

Pathological type	Histopathological changes	Degree	Scores
Steatosis	Indicating the normal state.	None	0
	The lesion was between 0 and 1 points.	±	0.5
	The fat vacuoles in the liver cells were small and scattered.	Minor lesions +	1
	The fat vacuoles in the liver cells are larger and wider in range.	Moderate lesions ++	2
	The fat vacuoles are fused into large vacuoles, and the nucleus is squeezed into the cell membrane. It likes the adipocyte.	Severe lesions +++	3

*^*a*^The pathological scores of fatty liver are divided into five grades (0–3), according to the levels of steatosis*.

**FIGURE 3:**
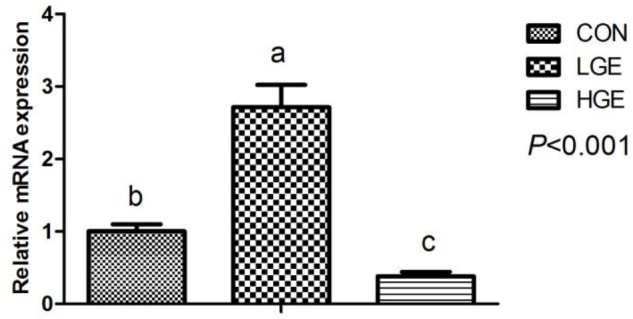
Effect of dietary GEN on GnRH mRNA expression in the hen hypothalamus (*n* = 8 replicates). Data represent the mean ± SEM of the values. Mean values without a common identifier (a, b, and c) differ significantly among the three groups (*P* < 0.05).

### Serum Metabolic Indices and Routine Blood Tests

The changes of serum metabolic indices are shown in Table 4. The serum levels of TC (*P* < 0.05), TG (*P* < 0.05), and FFA (*P* = 0.07) in LGE and HGE group were significantly reduced compared with the CON group, indicating GEN treatment improved the disorder of lipid metabolism. The high-GEN treatment significantly decreased the serum GPT (*P* = 0.069) and CRE levels (*P* < 0.05), indicating that GEN treatment improved the liver and kidney functions. The function of VLDL is to transport TGs and TC to tissues. GEN treatment significantly increased the level of VLDL in the serum of laying hens (*P* < 0.05). RBC (*P* < 0.05), PLT (*P* = 0.053), PCT (*P* < 0.05), and WBC (*P* = 0.070) in the venous blood of HGE group was increased compared with levels in the CON group. The number of BASO in the blood of LGE group was increased compared with that in the CON group (*P* < 0.05). Additionally, GEN treatment reduced the number of LUC in blood of hens, indicating that GEN could reducing inflammatory reaction in the FLS hens.

### Antibody Titer in the Serum

As shown in Table 6, adding GEN to the diet of FLS hens during the late egg-laying period had no significant effect on the serum antibody titer.

**Table 6:** Effect of dietary GEN on serum antibody titers in hens.

Treatment	ND	RE-6	RE-7	RE-8	H9
CON	8.9 ± 0.4	9.8 ± 0.7	8.4 ± 0.5	7.1 ± 1.2	11.4 ± 0.7
LGE	9.4 ± 0.8	10.0 ± 1.0	8.6 ± 0.8	6.7 ± 0.8	12.0 ± 0.8
HGE	9.0 ± 1.0	10.1 ± 0.7	8.4 ± 1.0	6.0 ± 1.2	11.9 ± 1.3
*P*-value	0.352	0.639	0.882	0.158	0.452

*ND, Newcastle disease; RE-6, RE-7, RE-8, and H9 (avian influenza). *n* = 9 replicates, 2 samples each replicate. Data represent the mean ± SD of values*.

### Measurement of Hepatic Antioxidant Indices

The effects of GEN on the antioxidant enzyme activity and MDA levels in the livers of the hens are shown in Table 7. Dietary GEN significantly decreased the level of MDA, a lipid peroxidation product, in the livers of FLS hens (*P* < 0.05). The activities of SOD in the livers of the LGE and HGE groups were increased by 10.58% (*P* = 0.184) and 37.35% (*P* < 0.01), respectively, compared with the activity in the CON group. Thus, dietary GEN enhanced the antioxidant capacity of FLS hens. However, GEN had no significant effects on the activity of hepatic CAT or GSH-Px.

**Table 7:** Hepatic antioxidant indices.

Treatment	T-SOD	CAT	GSH-Px	MDA
CON	157.25 ± 24.35b	18.23 ± 5.44	22.27 ± 2.15	0.53 ± 0.20a
LGE	173.88 ± 23.24b	21.98 ± 4.24	18.78 ± 3.50	0.33 ± 0.11b
HGE	215.93 ± 37.45a	17.41 ± 1.38	17.32 ± 6.15	0.36 ± 0.09b
*P*-value	0.002	0.111	0.210	0.023

*T–SOD, total superoxide dismutase (U/mg protein); CAT, catalase (U/mg protein); GSH-Px, glutathione peroxidase (U/mg protein); MDA, malondialdehyde (nmol/mg protein). *n* = 9 replicates, 2 samples each replicate. Data represent the mean ± SD of values. Mean values without a common identifier (a and b) differ significantly among the three groups (*P* < 0.05)*.

### Measurement of Liver Fatty Acid Profiles

As shown in Table 8, GEN treatment significantly decreased the total LSFA level (*P* < 0.05) and increased the C22:0 level (*P* < 0.01) compared with those of the CON group. GEN treatment increased the total n-3 family LCFAs in the livers of the HLS hens (*P* < 0.05) and decreased the C18:2n6c (*P* = 0.001) and C18:3n6 levels (*P* < 0.05). However, C18:3n3 was significantly less abundant in the livers of the LGE and HGE groups than in those of the CON group (*P* < 0.01). In addition, the levels of C20:3n6 and C20:4n6 in the livers of LGE (*P* < 0.05) and HGE (*P* < 0.01) hens were increased after GEN treatment compared with the levels in the CON group. On the whole, dietary GEN significantly decreased LCFAs (saturated, n-6 family, MUFAs and PUFAs; *P* < 0.01 for each category).

**Table 8:** Effects of GEN on long-chain fatty acids and triglyceride in the liver of hens.

	CON	LGE	HGE	*P*-value
LSFAs				
C12:0	0.090 ± 0.016a	0.042 ± 0.002b	0.056 ± 0.009b	<0.001
C14:0	2.03 ± 0.76a	0.46 ± 0.23b	0.79 ± 0.16b	<0.001
C15:0	0.20 ± 0.05a	0.08 ± 0.01b	0.13 ± 0.03b	0.001
C16:0	119.1 ± 30.5a	41.8 ± 11.8b	61.0 ± 7.5b	<0.001
C17:0	1.11 ± 0.27a	0.41 ± 0.05b	0.64 ± 0.13b	<0.001
C18:0	71.6 ± 21.5a	27.8 ± 4.6b	39.1 ± 6.2b	0.001
C20:0	0.24 ± 0.05a	0.18 ± 0.01b	0.19 ± 0.02b	0.019
C21:0	0.47 ± 0.04b	0.66 ± 0.08a	0.55 ± 0.04b	0.001
C22:0	0.23 ± 0.03b	0.4 ± 0.09a	0.33 ± 0.04a	0.002
C23:0	0.06 ± 0.03b	0.12 ± 0.02a	0.09 ± 0.01b	0.006
C24:0	0.21 ± 0.04	0.38 ± 0.09	0.37 ± 0.08	0.005
Monounsaturated				
C14:1	0.15 ± 0.06a	0.02 ± 0.03b	0.05 ± 0.02b	0.001
C16:1	6.88 ± 2.02a	1.57 ± 1.00b	2.55 ± 0.69b	<0.001
C18:1n9c	182.3 ± 50.2a	46.4 ± 18.6b	75.1 ± 15.6b	<0.001
C20:1	0.53 ± 0.11a	0.27 ± 0.08b	0.27 ± 0.06b	0.001
C22:1n9	0.021 ± 0.012	0.025 ± 0.002	0.024 ± 0.003	0.76
C24:1	0.16 ± 0.05	0.21 ± 0.03	0.18 ± 0.04	0.183
n-3 family				
C18:3n3	1.32 ± 0.23a	0.68 ± 0.28b	1.14 ± 0.27b	0.006
C20:5n3	0.00 ± 0.00	0.01 ± 0.01	0.01 ± 0.01	0.287
C22:6n3	1.89 ± 0.39b	2.73 ± 0.32a	2.75 ± 0.42a	0.005
n-6 family				
C18:2n6c	56.7 ± 11.6a	29.7 ± 5.7b	40.8 ± 5.7b	0.001
C18:3n6	0.43 ± 0.21a	0.16 ± 0.04b	0.28 ± 0.04b	0.017
C20:3n6	0.45 ± 0.11b	0.65 ± 0.04a	0.65 ± 0.05a	0.002
C20:4n6	5.62 ± 1.65b	8.32 ± 1.47a	7.99 ± 0.68a	0.015
n-3 family	3.20 ± 0.39b	3.49 ± 0.15b	3.90 ± 0.42a	0.022
n-6 family	63.15 ± 10.23a	38.86 ± 4.65b	49.73 ± 5.07b	0.001
n-6:n-3 PUFAs	18.63 ± 2.74a	11.40 ± 1.24b	12.88 ± 2.30b	<0.001
LSFAs	195.33 ± 52.94a	72.29 ± 16.44b	103.26 ± 13.11b	<0.001
MUFAs	190.04 ± 52.01a	48.45 ± 19.63b	78.19 ± 16.26b	<0.001
PUFAs	65.71 ± 11.34a	42.27 ± 4.74c	53.63 ± 4.95b	<0.001
LCFAs	452.2 ± 113.7a	163.00 ± 38.88b	235.0 ± 29.4b	<0.001
TG	428.9 ± 122.8	152.8 ± 67.7	244.6 ± 101.4	<0.001

*Data represent the mean ± SD of values obtained from six liver samples in each group (n = 6 replicates, mg/g, dried liver weight). Mean values without a common identifier (a and b) differ significantly among the three groups (*P* < 0.05). LSFAs, long-chain saturated fatty acids. LCFAs, long-chain fatty acids. TG, triglyceride*.

### The Effects of GEN on Metabolism-Regulating Factors in the Liver and Abdominal Fat

**FIGURE 4:**
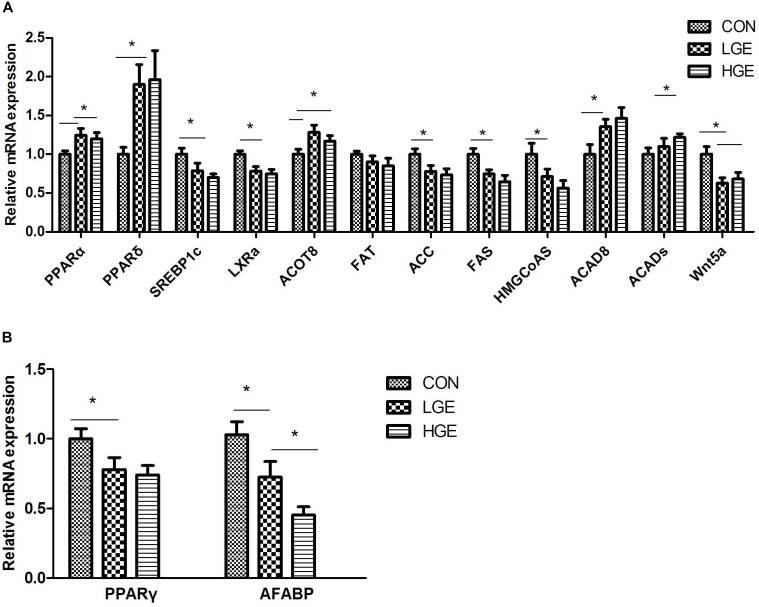
**(A,B)** Represent the effect of GEN supplementation on the relative mRNA expression of genes related to lipid metabolism in the liver and abdominal fat, respectively; *n* = 8 replicates. Data represent the mean ± SEM of the values. Means with ^∗^ are significantly different.

The liver is the main site of fat metabolism in poultry. The expression levels of genes relating to lipid metabolism in the chicken liver (Figure 4A) were examined. Dietary GEN significantly upregulated PPARα and PPARδ mRNA expression (*P* < 0.05). LXRα and SREBP1c are downstream signaling proteins of PPARs and are involved in fatty acid and cholesterol anabolism. The transcriptional levels of the genes encoding LXRα and SREBP1c were significantly reduced by both high- and low-dose GEN treatment with the downregulated expression of ACC, FAS, and 3-hydroxy-3-methylglutaryl-coenzyme A synthase (HMGCoAS). Acyl-CoA thioesterase (ACOT) 8 and acyl-CoA dehydrogenase (ACAD) 8, which are involved in β-oxidation, were more abundantly transcribed in the livers of the LGE and HGE groups than in those of the CON group (*P* < 0.05). GEN treatment and 400 mg/kg diet significantly upregulated the mRNA expression of ACADs in the liver compared with the expression in the CON group (*P* < 0.05). Additionally, GEN treatment reduced the transcriptional level of WNT5a and FAT in the liver (*P* < 0.05). Abdominal fat is one of the storage sites for lipids in poultry, especially when FLS occurs. PPARG is the gene encoding PPARγ, which promotes adipocyte differentiation and fat deposition. One of the downstream target genes regulated by PPARγ is adipocyte fatty acid-binding protein (AFABP). PCR results showed that the mRNA expression levels of PPARD and AFABP were significantly higher in the abdominal fat of the LGE and HGE groups than in those of the CON group (*P* < 0.05, Figure 4B).

### The Effects of GEN on Immunity-Regulating Factors in the Liver and Abdominal Fat

**FIGURE 5:**
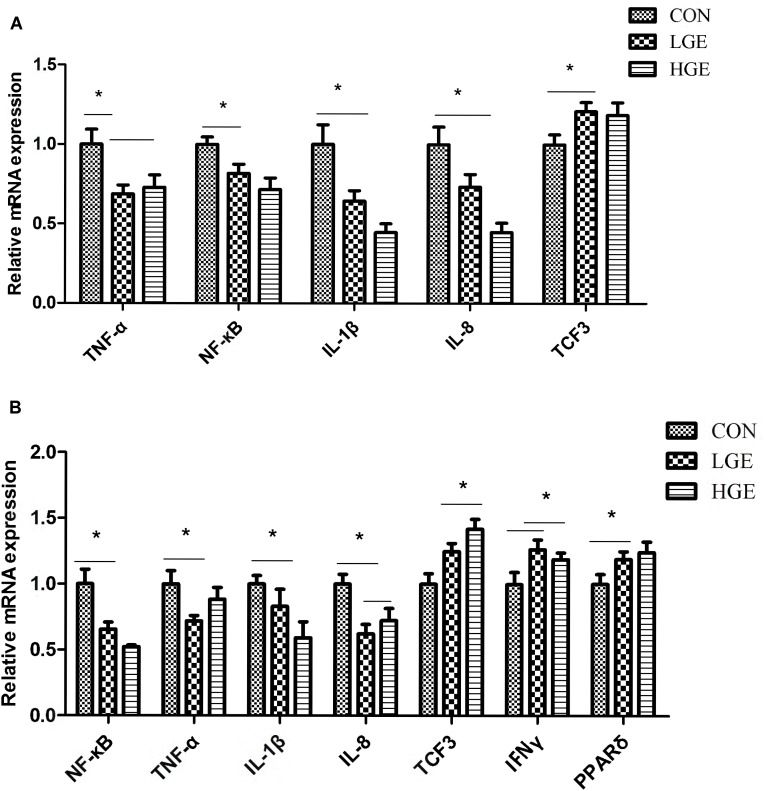
**(A,B)** Represent the effect of GEN supplementation on the relative mRNA expression of genes related to the inflammatory reaction in the liver and spleen, respectively, *n* = 8 replicates. Data represent the mean ± SEM of the values. Means with ^∗^ are significantly different.

In addition to hepatocytes, the liver tissue also contains sinusoidal endothelial cells, stellate cells, dendritic cells, Kupffer cells, and lymphocytes. These cells secrete a series of cytokines, which play an important role in immune regulation. The spleen is the main peripheral immune organ of poultry. As shown in Figure 5A, NF-κB, TNF-α, IL-8, and IL-1β mRNA expression was significantly upregulated in the livers and spleens of the LGE and HGE groups compared to expression in those of the CON group (*P* < 0.05). The results showed that GEN treatment significantly increased the transcription of TCF3 in the liver and spleen (Figure 5B, *P* < 0.05). Additionally, GEN treatment significantly increased the transcriptional levels of PPARδ and IFNγ (Figure 5B, *P* < 0.05).

## Discussion

The FLS has been reported to be one of the most fatal non-communicable diseases in layers (Mete et al., 2013). FLS is characterized by a large amount of fat deposited in the hepatocytes, resulting in fatty liver, liver hemorrhage, and obesity. Choline can bind to fat and prevent the abnormal accumulation of fat in the liver. In the present study, laying hens were fed the HELC diet as their basal diet to establish a model of FLS. Clinicopathological evaluation showed that the livers of layers fed with the HELC diet were pale yellow, friable, and swollen, with hemorrhagic spots and attached fat on the surface. Histopathological analysis demonstrated that the livers of the CON group had the typical FLS features of hepatocyte steatosis and altered hepatocyte shape. FLS has been reported to significantly reduce the egg production and feed intake of layers (Jiang et al., 2013). The antioxidant and estrogenic effects of GEN are stronger than those of other kinds of soybean ISOs. In the present study, 40 mg/kg GEN treatment significantly increased the laying rate of FSL layers during the late egg-laying period. However, 400 mg/kg GEN treatment significantly reduced the laying rate. Neither high- nor low-dose GEN supplementation in feed had any significant effect on feed intake or egg weight. Research has shown that an appropriate amount of daidzein, which is a type of ISO, can improve the reproductive performance of female Zhedong White geese by regulating reproductive hormones in the HPG axis (Zhao et al., 2013). In the current study, 40 mg/kg GEN treatment significantly increased the transcriptional level of GnRH in the hypothalamus of layers, while the same hormone was downregulated by 400 mg/kg GEN treatment. These effects on GnRH could explain the difference in the reproductive performance of FLS layers after the two levels of GEN treatment. In addition, the negative effects of high-dose GEN might also be due to the inhibition of tyrosine protein kinases and related effects on the physiological state of FLS layers. Studies on rodents also indicate that the effects of GEN on production performance vary with sex, age, endogenous hormone levels, and the GEN dose (Naaz et al., 2003; Penza et al., 2006; Castellano et al., 2011). GEN supplementation also upregulates ESRα and ESRβ mRNA expression in the livers of mice fed a high-fat diet (Tang et al., 2015). In the present study, low-dose GEN treatment improved the serum level of E2 in FLS layers, while high-dose GEN had no significant effect on the E2 level. Our work demonstrates for the first time that the effects of high and low doses of GEN on reproductive hormones, leading to significant differences in the production performance of hens with FLS.

Long-chain fatty acids and TGs accumulate in the liver when their synthesis, transport, and catabolism are disordered, which can cause hepatic steatosis (Koteish and Diehl, 2001; Bradbury and Berk, 2004). In the present study, the content of LCFAs in the livers of FLS hens was significantly reduced by GEN supplementation. Excessive intake and accumulation of MUFAs in the liver contributed to the pathological changes (Øyvind, 1991). We found that dietary GEN significantly reduced the content of MUFAs in the livers of FLS layers. The balance between n-6 and n-3 PUFAs is essential for maintaining metabolism and immunity in the body (El-Badry et al., 2007). n-3 PUFAs have potent anti-inflammatory, anti-proliferative, and immunoregulatory effects (Chapkin et al., 2007). n-3 PUFAs can inhibit fatty acid synthesis in the liver (Sekiya et al., 2003). In the present study, GEN treatment significantly increased total n-3 PUFAs in the livers of FLS layers, and the levels of C20:5n3 and C22:6n3 were significantly increased as well. According to a study by Araya et al., the ratio of n-6 to n-3 PUFAs is significantly increased in the hepatic phospholipid profiles of FLS patients compared with those of normal individuals (Araya et al., 2004). In our study, GEN treatment significantly reduced the content of n-6 LCFAs and the ratio of n-6 to n-3 PUFAs in the livers of FLS layers. However, we found that the levels of C20:3n6 and C20:4n6 (arachidonic acid, ARA) were significantly higher in the livers of the LGE and HGE groups than in those of the CON group. Dietary ARA, especially at moderate levels (0.22–0.56% dry weight), significantly enhanced growth and immune responses and modified the chemical composition of the whole body and liver of the juvenile Japanese seabass (Xu et al., 2010). However, the regulatory mechanism through which GEN influences PUFA levels remains to be studied further. This report is the first to clarify the change in the fatty acid profiles in the livers of laying hens after GEN treatment. Lipid metabolism operates differently in the actively laying hen than in many other animals. Accordingly, high- and low-dose GEN in feed significantly reduced the liver TG content and hepatic scores of layers. Additionally, the levels of TG and FFAs in the serum of hens in the LGE and HGE groups were significantly decreased after GEN treatment compared with the levels in the CON group, which could indicate that the disorder of lipid metabolism was significantly improved. Lipids in poultry plasma are transported into the extrahepatic tissue by binding to VLDL. Studies have shown that mutant apolipoprotein B (ApoB, the structural protein of VLDL) or MTP (responsible for recruitment of TGs) can cause hepatic steatosis by disrupting VLDL assembly (BerriotVaroqueaux et al., 2000; Tanoli et al., 2004). It was found in the present study that dietary GEN significantly increased the serum level of VLDL, which could reduce lipid accumulation in the liver. This result is consistent with the findings that GEN (0.5 g/kg diet) can alleviate the liver steatosis, cholesterolemia, and obesity symptoms induced by a high-energy diet in mice (Park et al., 2013). The liver and kidneys play important roles in the lipid metabolism of layers, and two relevant parameters (serum GPT and serum CRE) are both decreased by GEN treatment. High- and low-dose GEN treatment showed the same effects on lipid metabolism in FLS layers. However, the effects of the two doses on serum E2, as mentioned above, were significantly different. Therefore, we speculated that the alleviation of FLS by GEN in layers is not regulated only through the E2 signaling pathway.

Genistein has been reported to activate PPARs, which in turn activate the expression of transcription factors related to insulin sensitivity regulation, adipogenesis, and blood pressure (Dang et al., 2003). GEN is reported to upregulate the expression of carnitine acyltransferase (in an ESR-dependent manner) and promote lipolysis in HepG2 cells, and antagonistic effects exist between ESR and PPAR (Bitto et al., 2009). In the present study, high- and low-dose GEN treatment significantly upregulated the transcription levels of PPARα and PPARδ in the livers of supplemented hens compared with controls. PPARα is involved in the regulation of mitochondrial β-oxidation and microsomal ω-oxidation, and it inhibits lipogenesis through LXRα and SREBP1c in the liver. Therefore, PPARα plays an important role in the occurrence and development of NAFLD (Pyper et al., 2010). LXRα can form a positive feedback loop with SREBP1c and enhance lipogenesis in the liver (Willy et al., 1995). In the present study, high and low doses of GEN significantly downregulated the expression of SREBP1c and LXRα, as well as the target genes ACC, FAS, and FAT. The activation of FAT can promote the uptake of FFAs into the liver, thereby inducing hepatocytic steatosis (Nakamura et al., 2007). Therefore, dietary GEN inhibited fatty acid synthesis in the livers of FLS hens through the PPAR–LXRα–SREBP1c–ACC/FAS/FAT pathway. Recent studies have shown that activation of PPARδ can reduce the hepatic fat content by increasing glucose catabolism and inhibiting SREBP1c activity in the liver and promoting β-oxidation in the muscles (Wagner et al., 2011). In the present study, we found that GEN treatment upregulated the expression of ACAD8, ACADs, ACOT8, and FAT. ACADs are a family of mitochondrial flavoenzymes that catalyze dehydrogenation steps of the α- and β-oxidation processes. ACOT8 encodes ACOT 8, which is involved in fatty acid oxidation (Hunt and Alexson, 2002). Studies have shown that ACOT is strongly regulated by PPARs, which can also inhibit fatty acid synthesis (Libertini and Smith, 1978; Miyazawa et al., 1981). Therefore, it is likely that dietary GEN inhibits fatty acid synthesis and enhances β–oxidation in the livers of layers through the PPAR–ACAD/ACOT and PPAR–LXRα–SREBP1c–ACC/FAS/FAT pathways, which would provide a mechanism for the GEN-linked changes in hepatic fatty acid profiles.

Peroxisome proliferator-activated receptorγ mainly promotes energy storage and adipocyte differentiation (Vosper et al., 2001). Upregulation of PPARγ has been reported to cause hepatic steatosis (Hoekstra et al., 2009). In the present study, GEN treatment significantly downregulated the transcriptional level of PPARγ in the abdominal fat and liver. Therefore, the abdominal fat rate and liver TG content of layers were decreased after GEN treatment. HMGCoAS is the key enzyme that catalyzes the *de novo* synthesis of cholesterol, which increases the fat content of the liver (Okey, 1933). A prior study showed that PPARα could inhibit expression of the gene encoding HMGCoAS (Fiorucci et al., 2010), and we found that dietary GEN also downregulated HMGCoAS mRNA expression in the liver. The levels of TC in the serum were significantly decreased after GEN treatment. AFABP, the target gene of PPARγ, promotes lipid deposition in adipocytes. In the present study, dietary supplementation with high and low doses of GEN significantly reduced the mRNA expression of AFABP in the abdominal fat of FLS layers. Thus, the abdominal fat rate was significantly decreased by GEN treatment. In addition, dietary GEN downregulated the expression of Wnt5a in the livers of FLS layers. GEN has been reported to inhibit the Wnt signaling pathway through epigenetic modification (Zhang et al., 2013). Interestingly, activation of the Wnt/β-catenin signaling pathway can inhibit β-oxidation through PPARs (Duval et al., 2004; Essers et al., 2005; Haluzík and Haluzík, 2006). Therefore, GEN might also indirectly activate PPARs through the Wnt signaling pathway, thus alleviating the fat accumulation typical in FLS hens. FFAs and the PPAR signaling pathway regulate each other. Studies have shown that PUFAs can activate PPARα to upregulate the transcription of genes involved in β-oxidation and downregulate the expression of genes involved in hepatic fat synthesis (Buang et al., 2004). PUFAs can also promote ApoB-100 secretion and enhance TG transport into the liver (Videla et al., 2004). In particular, n-3 PUFAs can increase insulin sensitivity and improve disordered lipid metabolism. Therefore, GEN treatment might further alleviate FLS through PUFAs.

Fatty acid accumulation is not only a characteristic feature of the FLS but also causes insulin resistance, lipid peroxidation, and an inflammatory response (Randle et al., 1963; Sotovaca et al., 2013). FFAs can stimulate the translocation of Bax into lysosomes, promote the release of cathepsin B into the cytoplasm, and then upregulate the expression of TNF-α through NF-κB. TNF-α can induce the accumulation of TG in the liver, leading to hepatocyte steatosis. TNF-α can also promote lysosomal permeability and activate NF-κB, thus perpetuating a vicious cycle of liver injury (Feldstein et al., 2004). Pathological analysis revealed inflammatory cell infiltration around the blood vessels in the fatty livers of layers fed with an HELC diet. TNF-α is the first pro-inflammatory factor released in the immune response (Zeng et al., 2014) and can also increase ROS in the liver and induce lipid peroxidation (Crews et al., 2006). In the present study, GEN treatment significantly increased SOD activity and the total antioxidant capacity in hens’ livers, which was consistent with the report that soybean ISOs could improve SOD activity in RBCs (Chen et al., 2004). MDA can interact with NF-κB to increase the release of TNF-α (Kishore et al., 2002). Our results showed that dietary GEN supplementation significantly reduced the level of MDA in the livers of FLS layers, reflecting alleviation of lipid peroxidative injury. Additionally, GEN treatment downregulated the expression of TNF-α and NF-κB in the livers and spleens of FLS layers. Therefore, the increased antioxidant capacity induced by GEN can relieve the inflammatory response in FLS layers. TNF-α can induce the release of IL-6 and IL-8 from Kupffer cells in the liver. It is reported that excessive deposition of palmitic acid in the liver can activate NF-κB and c-Jun N-terminal kinase-1 (JNK-1) and increase the IL-8 level, leading to an inflammatory reaction (Joshibarve et al., 2007). n-3 PUFAs in the liver have been suggested to alleviate the inflammatory response in obese mice (Hamamoto et al., 1993). In our study, the content of LSFAs (specifically, palmitic acid) in the liver was reduced and the content of n-3 PUFAs was increased by GEN treatment. The mRNA expression of pro-inflammatory factors (IL-8, IL-1β) in the liver and spleen was also decreased after GEN treatment, as were the organ indices. The deficiency of IL-1β and inflammatory corpuscle components could impede the inflammatory response induced by the fatty liver (Kamari et al., 2011; Vandanmagsar et al., 2011). From routine blood tests and serum biochemical indices, we can see that a high dose of GEN increased the RBC, PLT, PCT, and WBC in the blood of FLS layers. Low-dose GEN treatment increased BASO and reduced LUC compared with the control values. These results further confirmed that the GEN treatment could alleviate the inflammatory response in FLS layers. We further examined the upstream regulatory genes of pro-inflammatory factors. Interesting, the expression levels of PPARδ in both liver and spleen tissue were downregulated after GEN treatment, which was consistent with the change in pro-inflammatory factors. It is reported that PPARδ can mitigate the inflammatory response by inhibiting NF-κB expression (Hoekstra et al., 2009). Similarly, activation of PPARδ can delay the production of inflammatory factors, such as TNF-α, at injury sites, regulate the adaptation of cells to stress, and prevent the apoptotic signals caused by inflammatory factors (Wahli, 2002). Therefore, dietary GEN can directly inhibit the NF–êB signaling pathway and decrease pro-inflammatory factors by activating PPARδ. Moreover, high and low doses of GEN significantly increased the transcription levels of IFNγ and TCF3 in the liver and spleen. It is well known that IFN-γ plays an important role in disease resistance and the immune response in chickens. E proteins, which belong to class I of the basic helix-loop-helix (bHLH) family of transcription factors, are encoded by the TCF3 gene, which is required for lymphocyte proliferation (Henthorn et al., 1990). The TCF3 gene can also inhibit the Wnt signaling pathway (Lluis et al., 2011). Therefore, dietary GEN might regulate inflammatory factors and the Wnt signaling pathway through TCF3, but the interactions between them remain to be studied further. In the present study, dietary GEN supplementation had no significant effects on antibody titers, perhaps because few immunizations were given to the hens during the late laying period. Dietary GEN supplementation indirectly alleviated the FLS in laying hens by improving the antioxidant capacity and regulating the fatty acid profile. In addition, the supplement directly relieved the inflammatory response by regulating the expression of inflammatory factors (IL-8, IL-1β, and IFNγ) *via* PPARδ and TCF3.

## Conclusion

In conclusion, GEN supplementation in feed inhibited fatty acid synthesis and enhanced β-oxidation in the livers of layers with FLS through the PPAR–ACAD/ACOT and PPAR–LXRα–SREBP1c–ACC/FAS/FAT pathways. Dietary GEN altered the liver fatty acid profile of FLS hens, thereby alleviating fat deposition and lipid metabolism disorder. GEN supplementation improved the antioxidant capacity of layers. We concluded that dietary GEN could indirectly help alleviate the FLS in laying hens by improving the antioxidant capacity and fatty acid profile. Additionally, GEN could directly alleviate the inflammatory response by regulating the expression of inflammatory factors (IL-8, IL-1β, and IFN-γ) *via* PPARδ and TCF3. GEN supplementation at 40 mg/kg of diet can significantly improve the laying rate of hens. This study provides a comprehensive analysis of the effects of dietary GEN on FLS hens from the perspectives of fatty acid metabolism, antioxidation, and the inflammatory response. The optimum dosage range of dietary GEN still needs to be clarified.

## Ethics Statement

This study was carried out in accordance with the recommendations of Animal Welfare Committee of China Agricultural University (CAU/No. 160515-2). The protocol was approved by the Animal Welfare Committee of China Agricultural University (CAU/No. 160515-2).

## Author Contributions

The research was designed and conducted by ZL and YG. The animal experiment was conducted by KX and GL. The detection and analysis works were conducted by ZL and DL. The writing was conducted by ZL independently.

## Conflict of Interest Statement

The authors declare that the research was conducted in the absence of any commercial or financial relationships that could be construed as a potential conflict of interest.

## Abbreviations

ACC: acetyl coenzyme A synthetase
CON: control group
E2: estrogen
FAS: fatty acid synthase
FAT: fatty acid transporter
FFAs: free fatty acids
FLS: fatty liver syndrome
GEN: genistein
GnRH: gonadotropin-releasing hormone
GPT: glutamic-pyruvic transaminase
HGE: high-genistein
HELC: high-energy and low-choline
IL: interleukin
LCFAs: long-chain fatty acids
LGE: low-genistein
LPL: lipoprotein lipase
LSFAs: long-chain saturated fatty acids
LXRα: liver X receptor alpha
MUFAs: monounsaturated fatty acids
NAFLD: non-alcoholic fatty liver disease
PPARs: peroxisome proliferator-activated receptors
PUFAs: polyunsaturated fatty acids
SREBP1c: sterol regulatory element-binding protein 1
TC: total cholesterol
TG: triglyceride
TNF-α: tumor necrosis factor alpha
